# Case report: A study on disease diagnosis and treatment outcome of a case of bilateral malignant solitary fibrous tumor of the kidneys

**DOI:** 10.3389/fonc.2025.1436015

**Published:** 2025-02-14

**Authors:** Yujie Cui, Bingjie Li, Jinlong Liang, Liya He, Hongzhen Zhang

**Affiliations:** ^1^ Oncology Department, Hebei General Hospital, Shijiazhuang, Hebei, China; ^2^ Pathology Department, Hebei General Hospital, Shijiazhuang, Hebei, China; ^3^ Graduate School of Hebei North College, Zhangjiakou, Hebei, China

**Keywords:** malignant solitary fibroma tumor, CD34, STAT6, paraneoplastic syndrome, therapy

## Abstract

Solitary fibrous tumor (SFT) is widely present in human connective tissues. It is mostly found in the pleura and other extrapleural sites. There is limited literature on malignant SFT in the retroperitoneum. In this study, we retrospectively reviewed the clinical data of a case of bilateral malignant SFT patient admitted to our hospital in May 2021.The patient had a huge tumor load and multiple bone metastases at the first diagnosis. Due to the need for lifelong hemodialysis after double nephrectomy and multiple bone metastases, the patient gave up surgical treatment, underwent drug treatment, and finally survived for 30 months.

## Introduction

Solitary fibrous tumor (SFT) is a rare spindle cell tumor that originates from dendritic interstitial cells expressing the CD34 antigen, accounting for less than 2% of all soft tissue tumors. First reported by Klemperer and Rabinz in 1931, SFT is predominantly found in human connective tissues, particularly in the pleura and other extrapleural sites. Despite its rarity, SFT has been increasingly recognized in recent years, with a growing body of literature focusing on its clinical presentation, pathological features, and management. However, literature on malignant SFT, especially in the retroperitoneum, remains limited. This study aims to contribute to the existing body of knowledge by presenting a detailed case report of a patient with bilateral malignant SFT, highlighting the diagnostic challenges and treatment outcomes. The main question addressed in this study is the clinical and pathological characteristics of bilateral malignant SFT, with a focus on the novelty of the case in terms of its presentation and management.

## Case presentation

**Figure 1:**
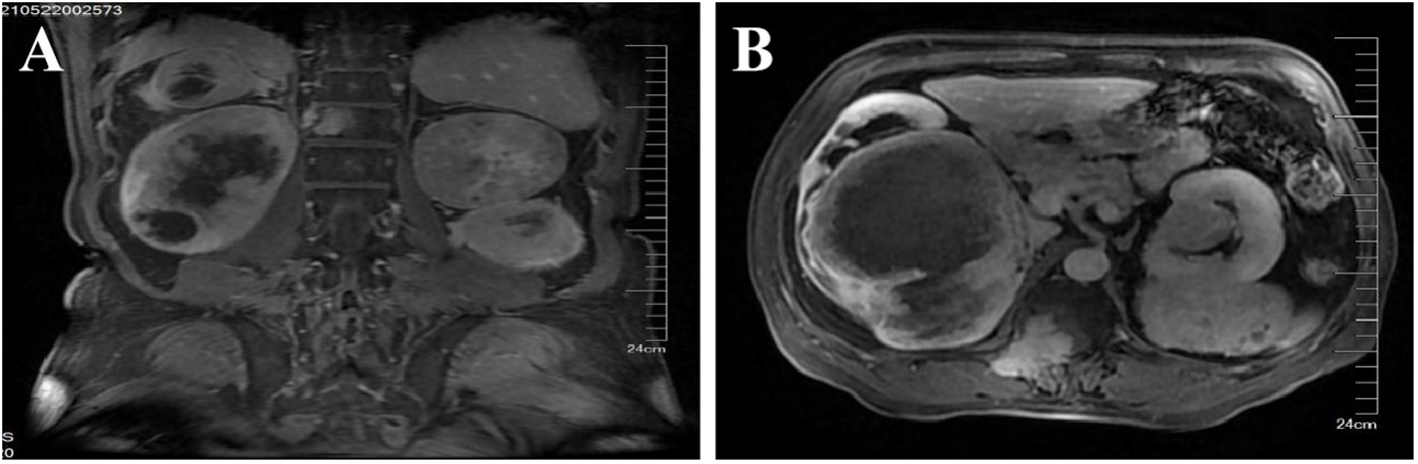
MRI of both kidneys. Both kidneys are enlarged in volume and irregular in shape, and multiple round and irregular masses can be seen, with uneven enhancement. The larger one is located in the right kidney, with a size of about 179 × 121 × 135 mm, and the bilateral renal pelvis is deformed by compression. Bone metastasis of chest 12, right adnexal area, and right sacrum may occur. **(A)** Sagittal position. **(B)** Coronal position.

**Figure 2:**
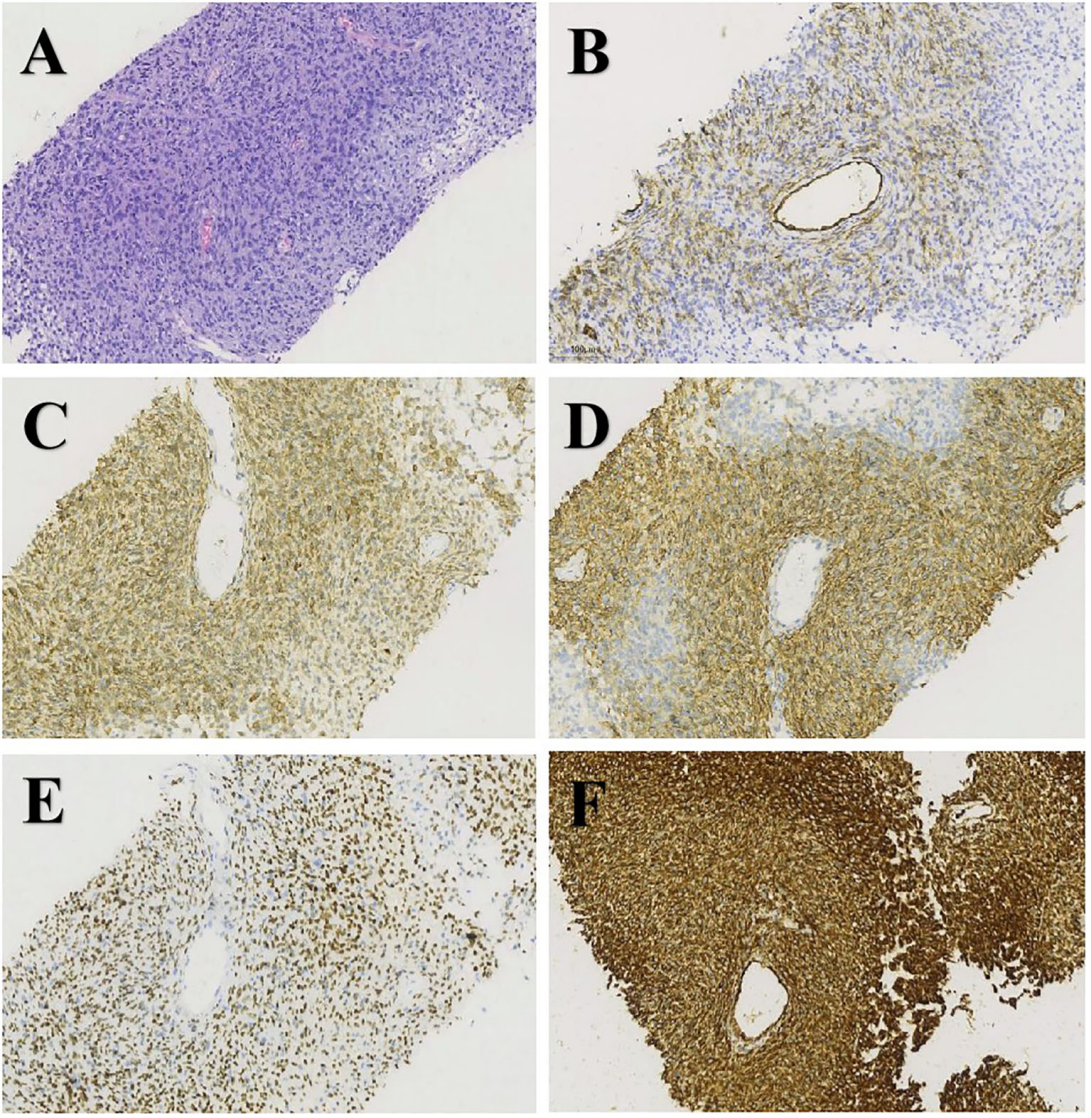
H&E staining and immunohistochemistry of biopsy specimens: **(A)** H&E (×200), **(B)** CD34 (×200), **(C)** Bcl-2 (×200), **(D)** CD99 (×200), **(E)** STAT6 (×200), and **(F)** vimentin (×200).

The patient, a 67-year-old man, was admitted to our hospital in May 21, 2021 due to low back pain. The physical examination results showed that a mass of about 20 cm × 10 cm was found in the right kidney area and a mass of about 5 cm × 5 cm was found in the left kidney area. The masses on both sides are hard, fixed, and have no obvious tenderness. There was mild tenderness in each vertebra. The tumor markers at the initial diagnosis of the patient were as follows—CEA: 3.64 ng/mL, CA19-9: 20.35 U/mL, and CA125: 18.42 U/mL, and no abnormalities were detected. The thoraco-abdominal pelvic CT image showed multiple space-occupying lesions in both renal areas as well as dilatation of the pelvis calyces and the upper ureter. Multiple low-density shadows can be seen in the left adnexa of T5, the right posterior part of the T8 vertebra, and the right adnexa of T12 and S1. Upon double renal MRI, it was noted that the volume of both kidneys increased and the shape was irregular, multiple round and irregular masses were seen, and the enhancement was uneven and enhanced. The larger one was located in th**e** right kidney, the size was about 179 × 121 × 135 mm, and the bilateral renal pelvis was deformed under pressure (**Figure 1A**). At the thoracic 12 and right adnexal region, sacrum partial right side bone metastasis is possible (**Figure 1B**). Based on the patient’s symptoms, signs, and imaging findings, the possible differential diagnosis of bilateral giant renal masses includes biphasic renal tumors, renal cell carcinoma, renal pelvis carcinoma, renal lymphoma, etc. To further clarify the diagnosis, ultrasound-guided percutaneous double kidney and sternal vertebra-occupying biopsy was performed on May 27, 2021, and the pathology combined with immunohistochemical staining (**Figures 2A–F**) was consistent with isolated fibrous tumors. The immunohistochemical staining results were as follows: CKpan (-), vimentin (+), EMA (partial +), Ki-67 (about 10%+), SSTR2 (-), S100 (-), HMB45 (-), desmin (-), SMA (partial +), PAX-8 (-), CD34 (partial +), P16 (partial +), MDM2 (-), PR (-), GFAP (-), CD99 (+), actin (-), STAT6 (+), and Bcl-2 (+). The final pathological diagnosis was SFT of the kidney with multiple bone metastases. In June 2021, sorafenib (400 mg bid) was given, and the condition was stable after regular reexamination. In October 2023, he was readmitted to the hospital due to recurrent hypoglycemic coma, considered paraneoplastic syndrome (non-islet cell neoplastic hypoglycemia), and received continued glucose pumping. A reexamination by abdominal CT showed that the mass of both kidneys was larger than before and the disease was progressing. On October 20, 2023 and November 11, 2023, he was treated with temozolomide 200 mg d1-2, 300 mg d3-7 + bevacizumab 300 mg d8 Q2w. The patient developed sudden abdominal pain with hematuria on December 18, 2023. Relevant laboratory tests showed bilateral renal tumor rupture, bleeding, and renal failure, for which he received hemodialysis, renal vascular embolization, and other treatments. His condition was critical, and he eventually died of a lung infection in December 2023.

## Discussion

### Epidemiology

SFT are typically diagnosed in adults over 40 years of age, with no significant difference observed between male and female individuals. Approximately 15% to 20% of SFTs exhibit invasive behavior, potentially involving both systemic soft tissues and the central nervous system (1). According to the 2013 WHO classification, soft tissue tumors characterized by excessive cellularity, active mitotic activity (>4 mitotic figures per 10 high-power fields), cellular atypia, necrosis, and/or marginal infiltrative growth are classified as malignant (2).

### Clinical presentation

Malignant retroperitoneal SFT often presents as painless abdominal masses, most of which have inert growth and generally have no obvious clinical symptoms. When the masses are large, they can press on adjacent structures and cause corresponding symptoms (3). Kidney SFT may have hematuria, back pain, back mass, and other symptoms. Moreover, SFT is associated with paraneoplastic syndromes, such as Doege–Potter syndrome and Pierre-Marie–Bamberger syndrome (4). These syndromes are systemic symptoms caused by biologically active substances secreted by tumors and are not related to direct invasion or metastasis of the tumor. Doege–Potter syndrome is mainly characterized by severe hypoglycemic symptoms, which are caused by the secretion of insulin-like growth factor II (IGF-II) by SFT. Pierre-Marie–Banberger syndrome is mainly characterized by white patches on the genital skin, which may be accompanied by itching or pain, and is caused by skin lesions due to abnormal immune system reactions triggered by SFT. The patient was admitted to the hospital due to the discovery of lumps in both kidney areas during a physical examination. Later on, symptoms such as hematuria, lower back pain, recurrent hypoglycemia, and itching of the skin around the genitals appeared, but no obvious white spot lesions were observed. It can be seen that the patient’s clinical manifestations are basically consistent with the abovementioned clinical characteristics.

### Differential diagnosis

The differential diagnosis for bilateral large renal masses includes biphasic renal tumors, renal cell carcinoma (RCC), urothelial carcinoma, and renal lymphoma. The biphasic tumors show imaging heterogeneity with irregular borders and may have necrosis and cystic changes; CK7 and CK20 are often positive (5). RCC, the most common renal malignancy, appears as irregular solid masses with heterogeneous enhancement on CT/MRI; PAX8, CAIX, and CD10 are positive. Urothelial carcinoma presents as masses in the renal pelvis or ureter, often with hydronephrosis and filling defects; CK7, CK20, and p53 are markers. Renal lymphoma features multiple low-density masses with mild to moderate enhancement; CD20, CD3, and BCL-2 are positive. Each condition has distinct symptoms and imaging characteristics (6).

### Pathological diagnosis

Pathological diagnosis is the gold standard for disease diagnosis. Typical immunohistochemical manifestations of SFT are being positive for CD34 and nuclear STAT6 and positive for other immune markers such as Bcl-2, vimentin, and CD99. CD34 and STAT6 are usually absent in some dedifferentiated SFT (7). Ki-67 >5% is conducive to the identification of benign and malignant SFT and is considered to be an independent factor in predicting malignant potential (8). In addition, the presence of NAB2–STAT6 fusion protein detected by molecular pathology can also indicate the diagnosis of SFT, especially in cases of atypical histopathological features (9). This fusion gene promotes tumor cell proliferation and survival by activating the STAT6 signaling pathway. In normal function, NAB2 and STAT6 are involved in transcriptional regulation, cell differentiation, and immune regulation, respectively. The detection of NAB2–STAT6 fusion gene not only helps diagnose SFT but also provides potential targets for prognosis evaluation and targeted therapy. The patient’s immunohistochemical results conform to the abovementioned description, but the patient did not undergo molecular pathological testing.

### Treatment and prognosis

At present, there are no standardized guidelines for the treatment of SFT, and multidisciplinary discussions are needed to find a better treatment plan (10). The main treatment is surgical resection, but it is prone to recurrence. Treatment for advanced and malignant cases is very limited due to poor response to conventional chemoradiotherapy, so research for systemic therapy is moving toward targeted agents and immune checkpoint inhibitors. At present, there are two main types of anti-angiogenic drugs: monoclonal antibodies (such as bevacizumab) (11) and tyrosine kinase inhibitors (such as sorafenib, pazopanib, regorafenib, etc.) (12–14). Secondly, immune checkpoint inhibitors (ICI) may have certain curative effects, but further large-scale clinical studies are needed (15, 16).

### Case-specific discussion

In the present case, the patient presented with a massive tumor burden and multiple bone metastases at the initial diagnosis. Given the need for lifelong hemodialysis following bilateral nephrectomy and the presence of multiple bone metastases, surgical treatment was deemed infeasible, and the patient underwent medical therapy. The patient was initially treated with sorafenib (400 mg bid), which stabilized the condition upon regular reexamination. However, the patient was readmitted in October 2023 due to recurrent hypoglycemic coma, which was considered a paraneoplastic syndrome (non-islet cell tumor hypoglycemia). A subsequent treatment with temozolomide and bevacizumab was initiated, but the patient’s condition progressed, with the renal masses increasing in size. The patient eventually succumbed to a lung infection in December 2023 after experiencing sudden abdominal pain with hematuria, which was attributed to bilateral renal tumor rupture and bleeding.

## Conclusion

In summary, malignant retroperitoneal solitary fibrous tumors (SFTs) are rare in clinical practice, and the existing literature primarily consists of retrospective studies on the diagnosis and treatment of SFT patients as well as related case reports. The clinical manifestations of these patients lack specific characteristics, making it essential for clinicians to perform pathological and immunohistochemical examinations to ensure accurate diagnosis and treatment, thereby minimizing the risk of misdiagnosis. Complete resection of the tumor is an effective method for the treatment of malignant retroperitoneal SFT, and adjuvant chemoradiotherapy is provided after surgery if necessary. The increasing focus on targeted therapies and immunosuppressants in the management of advanced SFT highlights the need for more standardized and prospective research to address the current gaps in this field.

## Data Availability

The original contributions presented in the study are included in the article/supplementary material. Further inquiries can be directed to the corresponding author.
